# Risk factors for prosthetic joint infections following total hip arthroplasty based on 33,337 hips in the Finnish Arthroplasty Register from 2014 to 2018

**DOI:** 10.1080/17453674.2021.1944529

**Published:** 2021-07-01

**Authors:** Valtteri J Panula, Kasperi J Alakylä, Mikko S Venäläinen, Jaason J Haapakoski, Antti P Eskelinen, Mikko J Manninen, Jukka S Kettunen, Ari-Pekka Puhto, Anna I Vasara, Laura L Elo, Keijo T Mäkelä

**Affiliations:** a Department of Orthopaedics and Traumatology, Turku University Hospital, and University of Turku, Turku;; b Turku Bioscience Centre, University of Turku and Åbo Akademi University, Turku;; c National Institute for Health and Welfare, Helsinki;; d Coxa Hospital for Joint Replacement, Tampere;; e Orton Hospital, Helsinki;; f Department of Orthopaedics and Traumatology, Kuopio University Hospital, Kuopio;; g Division of Operative Care, Department of Orthopaedic and Trauma Surgery, Oulu University Hospital, Oulu;; h Helsinki University Hospital, Helsinki, Finland

## Abstract

Background and purpose — Periprosthetic joint infection (PJI) is a devastating complication and more information on risk factors for PJI is required to find measures to prevent infections. Therefore, we assessed risk factors for PJI after primary total hip arthroplasty (THA) in a large patient cohort.

Patients and methods — We analyzed 33,337 primary THAs performed between May 2014 and January 2018 based on the Finnish Arthroplasty Register (FAR). Cox proportional hazards regression was used to estimate hazard ratios with 95% confidence intervals (CI) for first PJI revision operation using 25 potential patient- and surgical-related risk factors as covariates.

Results — 350 primary THAs were revised for the first time due to PJI during the study period. The hazard ratios for PJI revision in multivariable analysis were 2.0 (CI 1.3–3.2) for ASA class II and 3.2 (2.0–5.1) for ASA class III–IV compared with ASA class I, 1.4 (1.1–1.7) for bleeding > 500 mL compared with < 500 mL, 0.4 (0.2–0.7) for ceramic-on-ceramic bearing couple compared with metal-on-polyethylene and for the first 3 postoperative weeks, 3.0 (1.6–5.6) for operation time of > 120 minutes compared with 45–59 minutes, and 2.6 (1.4–4.9) for simultaneous bilateral operation. In the univariable analysis, hazard ratios for PJI revision were 2.3 (1.7–3.3) for BMI of 31–35 and 5.0 (3.5–7.1) for BMI of > 35 compared with patients with BMI of 21–25.

Interpretation — We found several modifiable risk factors associated with increased PJI revision risk after THA to which special attention should be paid preoperatively. In particular, high BMI may be an even more prominent risk factor for PJI than previously assessed.

Prosthetic joint infection (PJI) is currently the most common reason for revision surgery after THA (FAR 2016). A prior study based on Nordic data from 1995 to 2009 stated that 0.6% of all THAs were revised due to deep infection and that the risk was increasing towards the end of the study period (Dale et al. 2012). Cumulative incidence of PJI after primary THA from 1998 to 2009 in Finland was 0.92% (Huotari et al. 2015). PJI is a devastating complication; it can lead to reduced physical functioning, pain, poor quality of life (Cahill et al. 2008, Moore et al. 2015), and at worst even death of the patient. Thus, it is important to know PJI risk factors to be able to reduce PJIs.

Risk factors for PJI can be divided to patient- and surgical-related factors. Previously known patient-related risk factors PJI include increased comorbidity, morbid obesity, male sex, and operative diagnosis, whereas long duration of operation is a surgery-related cause for infection (Pedersen et al. 2010, Kong et al. 2017, Smith et al. 2018). Risk factors for PJI after primary THA have not been previously assessed based on FAR data. FAR data contents were thoroughly updated in 2014 to include parameters such as BMI, ASA class, and duration of surgery (FAR 2016). We determined the risk factors for first PJI revision after primary THA.

## Patients and methods

FAR was established in 1980 and since then it has been compiling data on arthroplasty surgery in Finland (Paavolainen et al. 1991). It is mandatory for all Finnish private and public healthcare units to provide information of arthroplasty surgery to the National Institute of Health and Welfare to maintain the FAR database (Puolakka et al. 2001). All Finnish citizens have a unique identification number that connects the person and the primary and possible revision THA. Reporting the patient- and surgery-related data to FAR is performed using a standard online sheet that is completed during the operation. Dates of death are obtained from the Population Register Centre. Currently over 95% of all primary THAs and 81% of all revisions performed are reported to FAR (FAR 2016).

Several new parameters were included to the FAR in May 2014. These were surgical approach, BMI, ASA class, intraoperative bleeding, duration of the operation, level of education of surgeon and assistant, mode of anesthesia, intraoperative complications, and previous operations on the same joint.

The following 25 risk factors were considered as covariates based on previously reported associations with PJI and prior clinical knowledge: age group (≤ 55, 56–65, 66–75, ≥ 76 years), sex, simultaneous bilateral operation (yes, no), ASA class (I, II, III–IV), BMI (≤ 20, 21–25, 26–30, 31–35, > 35), diagnosis (primary osteoarthritis, fracture, inflammatory arthritis, other), hospital volume (low [< 240 THAs performed annually], medium [240–480], high [> 480]), level of education of the surgeon (specialist, resident), level of education of the first assistant (specialist, resident, other), surgical approach (posterior, anterolateral, anterior), bleeding (< 500mL, > 500mL), duration of the operation (< 45, 45–59, 60–89, 90–120, > 120 minutes), anesthesia mode (spinal, epidural, general, nerve block), local infiltrative anesthesia (LIA) (yes, no), perioperative complication during operation (no complication, calcar fracture, trochanteric fracture, femoral shaft fracture, acetabular fracture), previous operation on the same joint such as osteotomy or osteosynthesis (yes, no), antibiotic prophylaxis (cefuroxime, clindamycin, vancomycin, other, not used), antithrombotic prophylaxis (enoxaparin, rivaroxaban, tinzaparin, warfarin, other, not used), anticoagulant medications (tranexamic acid, no, other), mechanical antithrombotic prophylaxis (calf muscle pump, surgical stocking, not used), antimicrobial incise drape (yes, no), fixation method (cementless, cemented, hybrid, reverse hybrid), bearing couple used (ceramic-on-ceramic, ceramic-on-ultra-highly cross-linked polyethylene (UHXLPE), metal-on-UHXLPE, ceramized metal-on-UHXLPE, other) and femoral head size (28, 32, 36, > 36 mm). In addition, we tested potential association of operated side (right, left) with revision for PJI.

We extracted data on 33,337 primary THAs and 350 revision operations due to PJI after the primary THA performed in Finland from May 2014 to January 2018 (Table 1, see Supplementary data). The survival endpoint was revision operation where at least 1 component was removed or exchanged due to PJI. Determining PJI as indication for revision operation was performed by the operating surgeon based on preoperative evaluation and clinical presentation. These evaluations should be based on recommended guidelines for diagnosing PJI (Parvizi et al. 2016). Unfortunately, FAR data contents do not include data on for example intraoperative bacterial cultures. Follow-up ranged between 0 and 3.7 years. The vast majority of PJI revisions (334 of 350) occurred during the first year after primary THA, but we decided to include the whole of the follow-up period and all of the cases. There were 2,839 patients with both hips operated (5,678 operations). In 456 patients both hips had been operated simultaneously. Bilateral THRs were treated as 2 independent observations, since bilaterality has been shown to have a negligible influence on the risk of revision for infection (Ranstam and Robertsson 2010). 2.4% of patients died during the study period. Although death can be considered as competing risk leading to potential overestimation of incidence of revision, we did not perform competing risk analysis, as our main focus was on the estimation of relative revision risks in which the Cox regression model has been reported to provide more accurate results (Ranstam and Robertsson 2017). Revisions performed for other reasons (fracture, dislocation etc.) were censored when they occurred.

**Table 2.** Univariable analysis of possible risk factors for revision for PJI

Variable	Hazard ratio (95% CI)
Sex (reference male)	
Female	0.6 (0.5–0.7)
ASA class (reference ASA I)	
ASA II	1.7 (1.1–2.7)
ASA III–IV	2.5 (1.6–3.9)
BMI (reference BMI 21–25)	
≤ 20	0.7 (0.2–2.1)
26–30	1.3 (0.9–1.8)
31–35	2.3 (1.7–3.3)
> 35	5.0 (3.5–7.1)
Preoperative diagnosis (reference primary osteoarthritis)	
Fracture	1.0 (0.6–1.7)
Inflammatory arthritis	1.3 (0.7–2.7)
Other	1.6 (1.1–2.2)
Intraoperative bleeding (reference < 500 mL)	
> 500 mL	1.5 (1.2–1.9)
Anesthesia (spinal)(reference no)	
Yes	0.6 (0.4–0.8)
Anesthesia (epidural)(reference no)	
Yes	2.2 (1.4–3.5)
Anesthesia (general)(reference no)	
Yes	1.7 (1.2–2.3)
Antithrombotic prophylaxis (reference enoxaparin)	
Warfarin	2.7 (0.9–8.4)
Rivaroxaban	0.8 (0.6–1.0)
Tinzaparin	0.6 (0.3–1.2)
Not used	2.8 (1.5–5.3)
Other	0.6 (0.3–1.2)
Bearing couple (reference metal-on-UHXLPE)	
Ceramic-on-ceramic	0.4 (0.2–0.7)
Ceramic-on-UHXLPE	0.9 (0.6–1.1)
Ceramized metal-on-UHXLPE	0.9 (0.5–1.5)
Other	0.1 (0.0–0.6)
Femoral head size (reference 32 mm)	
28 mm	2.8 (1.2–6.5)
36 mm	1.9 (1.4–2.6)
> 36 mm	2.1 (0.7–5.7)

UHXLPE = ultra-highly crosslinked polyethylene.

### Statistics

**Figure 1.**
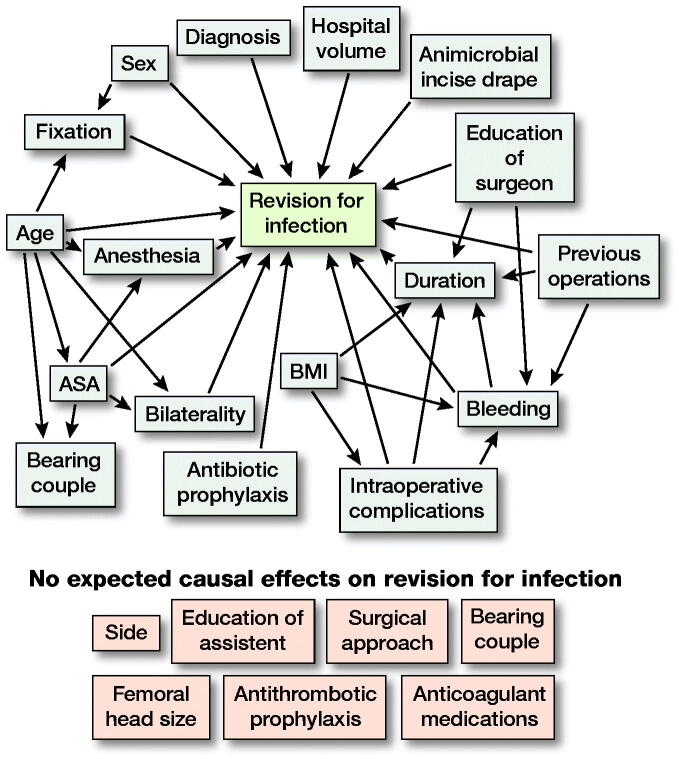
A directed acyclic graph (DAG) was constructed under the following assumptions: 1. THA “revision for infection” is dependent on “patient age,” “sex,” ‘bilaterality,” “ASA class,” “BMI,” “diagnosis,” “hospital volume,” “education of surgery,” “bleeding,” “duration,” “intraoperative complications,” “previous operations,” “antimicrobial incise drape,” “anesthesia,” “antibiotic prophylaxis,” and type of THA “fixation.” Choice of “side,” “education of assistant,” “surgical approach,” “bearing couple,” antithrombotic prophylaxis,” “anticoagulant medications,” and “femoral head size” are not expected to affect “revision for infection” due to clinical suspicion. 2. “Fixation” is dependent on “age” and “sex” because older and female patients have probably received a cemented or hybrid THA due to their poorer bone quality. “Bearing couple” may be dependent on age because surgeons have probably chosen ceramic-on-ceramic bearing couple in younger patients. “Bearing couple” may also be dependent on ASA class for the same reason. ASA class is partly dependent on age by definition. “Bilaterality” is dependent on “age” and “ASA class” because both hips are seldom operated on in elderly or high ASA class patients. 3. “BMI” may be affected by “duration” and “intraoperative complications” due to more difficult operation with high BMI. “Duration” may be dependent on “education of surgery” due to experience factor. “Bleeding,” “duration,” and “previous operations” may be dependent on clinical basis. 4.“Anesthesia” is dependent on “ASA class” and “age” because general anesthesia is usually avoided in elderly patients.

The unadjusted rate for revision due to PJI with 95% confidence intervals (CI) was first estimated with Kaplan–Meier analysis. Then univariable and multivariable Cox proportional hazards regression models were used for estimation of possible risk factors and hazard ratios with CIs for first infection revision operation (Tables 2 and 4, and Table 3, see Supplementary data). We performed a directed acyclic graph (DAG) analysis (Figure 1) based on the previous medical literature and the clinical practice to organize variables according to their supposed relation to PJI revision and to other variables. For all the variables in the univariable analysis with potential confounding bias, we performed multivariable analysis by choosing the adjusting variables based on the DAG. In the multivariable analysis the following 8 risk factors were adjusted with associated covariates identified in DAG: ASA class (adjusted for age), intraoperative bleeding (adjusted for BMI, previous contributing operations, complications during surgery and level of education [surgeon]), duration of operation (adjusted for previous contributing operations, level of education [surgeon], intraoperative bleeding, BMI and complications during surgery), anesthesia mode (adjusted for age and ASA class), bearing couple (adjusted for age and ASA class), fixation (adjusted for sex and age), simultaneous bilateral operation (adjusted for age and ASA class), and complications during the surgery (adjusted for BMI) (Tables 4 and 5).

**Table 4.** Multivariable analysis for revision for PJI

Variable	Hazard ratio (95% CI)
ASA class (reference ASA I)	
ASA II	2.0 (1.3–3.2)
ASA III–IV	3.2 (2.0–5.1)
Intraoperative bleeding (reference < 500 mL)	
> 500 mL	1.4 (1.1–1.7)
Anesthesia (spinal)(reference no)	
Yes	0.6 (0.4–0.8)
Anesthesia (epidural)(reference no)	
Yes	2.1 (1.3–3.4)
Anesthesia (general)(reference no)	
Yes	1.6 (1.2–2.3)
Bearing couple (reference metal-on-UHXLPE)	
Ceramic-on-ceramic	0.4 (0.2–0.7)
Ceramic-on-UHXLPE	0.9 (0.7–1.2)
Ceramized metal-on-UHXLPE	0.9 (0.5–1.6)
Other	0.1 (0.0–0.6)
Fixation (reference cementless)	
Cemented	1.1 (0.7–1.7)
Hybrid	1.3 (0.9–1.7)
Reverse hybrid	0.9 (0.5–1.5)

UHXLPE = ultra-highly crosslinked polyethylene.

ASA class was adjusted for age. Intraoperative bleeding was adjusted for BMI, previous contributing operations, complications during surgery, and level of education (surgeon). Spinal, epidural, and general anesthesia and bearing couples were adjusted for age and ASA class. Fixation was adjusted for sex and age.

**Table 5.** Uni- and multivariable analyses divided to suitable time intervals for the duration, simultaneous bilateral operation, anesthesia (LIA), and complications during surgery (fracture) due to not fulfilling the assumption of proportional hazards

	Univariable hazard ratio (95% CI)	Multivariable hazard ratio (95% CI)
Duration (minutes)(reference 45–59)		
Time interval 0–3 weeks		
< 45	1.0 (0.5–2.3)	1.1 (0.5–2.5)
60–89	1.4 (0.8–2.3)	1.3 (0.8–2.2)
90–120	1.4 (0.8–2.5)	1.3 (0.7–2.3)
> 120	3.3 (1.8–6.0)	3.0 (1.6–5.6)
Time interval > 3 weeks		
< 45	1.2 (0.4–3.7)	1.1 (0.3–3.4)
60–89	1.1 (0.5–2.2)	1.0 (0.5–2.2)
90–120	1.4 (0.6–3.1)	1.4 (0.6–3.1)
> 120	0.6 (0.2–1.5)	0.5 (0.2–1.4)
Simultaneous bilateral operation		
Time interval		
0–3 weeks	2.2 (1.2–4.2)	2.6 (1.4–4.9)
> 3 weeks	0.3 (0.07–1.0)	0.3 (0.07–1.0)
Anesthesia (LIA)		
Time interval		
0–3 weeks	0.7 (0.5–1.1)	0.7 (0.5–1.1)
> 3 weeks	1.5 (0.9–2.6)	1.5 (0.8–2.5)
Complications during surgery (fracture)		
Time interval		
0–5 weeks	0.3 (0.04–2.2)	0.4 (0.05–2.6)
> 5 weeks	8.8 (0.9–86.2)	8.6 (0.9–84)

In the multivariable analysis simultaneous bilateral operations and local infiltrative anesthesia were adjusted for age and ASA classification. Complication during surgery (fracture) was adjusted for BMI. Duration was adjusted for previous contributing operations, level of education (surgeon), intraoperative bleeding, BMI, and complications during surgery.

The proportional hazards assumption for Cox models were assessed from Kaplan–Meier curves graphically and by a statistical test based on scaled Schoenfeld residuals (Grambsch and Therneau 1994, Ranstam et al. 2011). A p-value < 0.05 for PH test indicated non-proportional hazards. In the multivariable analysis duration of the surgery > 120 minutes did not fulfil the proportional hazard assumption. Furthermore, LIA, simultaneous bilateral operation, and complication during surgery did not fulfil the assumption of proportional hazards in the univariable analysis. Therefore, we divided the follow-up time of these variables into suitable time intervals based on Kaplan–Meier analyses and performed uni- and multivariable analyses for these variables separately (Table 5).

All the statistical analyses were carried out using R statistical computing environment version 3.4.1 (R Foundation for Statistical Computing, Vienna, Austria. URL https://www.R-project.org/). A p-value < 0.05 was set as level of significance.

### Ethics, funding, and potential conflict of interest

Ethical approval: Dnro THL/506/5.05.00/2016.

Funding statement: This research received no specific grant from any funding agency in the public, commercial, or not-for-profit sectors. MSV reports funding from the Academy of Finland [grant number 322123]. The funders had no role in study design, data collection and analysis, decision to publish, or preparation of the manuscript.

The authors declare no conflicts of interest.

## Results

Most of the patients belonged to the age group from 66 to 75 years (37%), were women (57%), had an ASA class II (49%), and had a BMI of 26–30 (41%). The majority of patients received THA due to primary osteoarthritis (87%) and most of the operations lasted 60–89 minutes (49%). Most of the patients were operated on using spinal anesthesia (92%) and received THA with cementless fixation (62%), metal-on-ultra-highly crosslinked polyethylene (UHXLPE) bearing couple (50%), and 36 mm femoral head size (74%) (Table 1, see Supplementary data).

The overall Kaplan–Meier probability of no PJI revision at the end of the study period with 0–3.7-year follow-up time was 98.8% (CI 98.7–98.9).

Patients with advanced ASA class were associated with increased risk of revision for PJI in both univariable analysis (ASA class II vs. ASA class I HR 1.7 [CI 1.1–2.7] and ASA class III–IV vs. ASA class I HR 2.5 [CI 1.6–3.9]) (Table 2 and Table 3, see Supplementary data) and in multivariable analysis (ASA class II vs. ASA class I HR 2.0 [CI 1.3–3.2] and ASA class III–IV vs. ASA class I HR 3.2 [CI 2.0–5.1]) (Table 4).

Intraoperative bleeding over 500 ml was associated with increased risk of revision for infection when compared to bleeding less than 500 ml in both univariable analysis (HR 1.5 [CI 1.2–1.9]) (Table 2 and Table 3, see Supplementary data) and in multivariable analysis (HR 1.4 [CI 1.1–1.7]) (Table 4).

We found a decreased risk of revision for infection in univariable analysis for the patients with ceramic-on-ceramic bearing couple and also for the other group of bearing couples (ceramic-on-ceramic vs. metal-on-UHXLPE HR 0.4 [CI 0.2–0.7] and other vs. metal-on-UHXLPE HR 0.1 [CI 0.0–0.6]) (Table 2 and Table 3, see Supplementary data). The same association with ceramic-on-ceramic and other bearing couples was also found in multivariable analysis (ceramic-on-ceramic vs. metal-on-UHXLPE HR 0.4 [CI 0.2–0.7] and other vs. metal-on-UHXLPE HR 0.1 [CI 0.0–0.6]) (Table 4).

The use of epidural and general anesthesia was associated with increased risk of revision for infection in both univariable analysis (HR 2.2 [CI 1.4–3.5] and HR 1.7 [CI 1.2–2.3], respectively) (Table 2 and Table 3, see Supplementary data) and in multivariable analysis (HR 2.1 [CI 1.3–3.4] and HR 1.6 [CI 1.2–2.3], respectively) (Table 3). The use of spinal anesthesia was associated with decreased risk of revision for infection in both univariable (HR 0.6 [CI 0.4–0.8]) (Table 2 and Table 3, see Supplementary data) and in multivariable analysis (HR 0.6 [CI 0.4–0.8]) (Table 4).

Solely in the univariable analysis did we find an increased risk of revision for infection for the following parameters: preoperative diagnosis (other) HR 1.6 (CI 1.1–2.2), high BMI (BMI 31–35 vs. BMI 21–25 HR 2.3 [CI 1.7–3.3] and BMI > 35 vs. BMI 21–25 HR 5.0 [CI 3.5–7.1]), high volume hospitals vs. low volume hospitals HR 1.3 (CI 1.0–1.7), previous contributing operations HR 1.8 (CI 1.0–3.2), antithrombotic prophylaxis not used HR 2.8 (CI 1.5–5.3), femoral head size 36 mm vs. 32 mm HR 1.9 (CI 1.4–2.6), and 28 mm vs. 32 mm heads HR 2.8 (CI 1.2–6.5). Females compared with males HR 0.6 (CI 0.5–0.7) were associated with decreased risk of revision for infection in the univariable analysis (Table 2 and Table 3, see Supplementary data).

Simultaneous bilateral operation was associated with increased risk of PJI for the first 3 postoperative weeks in both univariable analysis HR 2.2 (CI 1.2–4.2) and in multivariable analysis HR 2.6 (CI 1.4–4.9) (Table 5). Further, duration of the operation over 120 minutes was associated with an increased risk of revision for infection for the first 3 postoperative weeks in both univariable analysis HR 3.3 (CI 1.8–6.0) and in multivariable analysis HR 3.0 (CI 1.6–5.6) (Table 5).

## Discussion

We found that high BMI, advanced ASA class, bleeding over 500 mL and the use of epidural and general anesthesia increased the risk of revision for PJI, whereas ceramic-on-ceramic bearing couple and spinal anesthesia decreased revision risk. Simultaneous bilateral operation and duration of operation over 120 minutes increased the risk of revision for PJI during the first 3 postoperative weeks. The cumulative rate of revision due to PJIs was 1.04%, which is slightly higher than published previously (Pedersen et al. 2010, Dale et al. 2011, 2012, Huotari et al. 2015, Kong et al. 2017, Lenguerrand et al. 2018). However, it is challenging to compare reported incidences of PJIs because of differences in definitions, time frame, surveillance systems, and completeness of reporting to registers (Wilson et al. 2007).

We found that high ASA class was associated with increased risk of revision due to PJI. ASA class is a crude estimate of a patient’s medical condition, and has been associated with PJI risk in numerous previous reports (Dale et al. 2011, Namba et al. 2012, Kong et al. 2017, Lenguerrand et al. 2018, Smith et al. 2018).

Another factor associated with increased risk of revision for infection in our multivariable analysis was bleeding over 500 mL. We are not aware of previous studies concerning intraoperative bleeding and PJI association, but blood transfusion and PJI have been associated previously (Kim et al. 2017). As intraoperative bleeding is a common indication for blood transfusion, we consider our finding to support the pre-existing evidence. In a comprehensive literature review it was stated that some association between intraoperative bleeding and PJI was found, but more quality studies are needed (Kwong et al. 2012).

Male sex was a risk factor for revision due to PJI in our study, which is in accordance with previous studies (Pedersen et al. 2010, Dale et al. 2012, Lenguerrand et al. 2018, Smith et al. 2018). Our data also supports the magnitude of risk presented previously (1.2–1.7-fold). Only 1 study has stated that female sex was associated with higher risk of revision for PJI (Namba et al. 2012). Reason for the increased PJI risk for males is not clear but may lie in confounding factors that are not included in the FAR such as smoking and alcohol abuse, both more common among males (WHO 2015, 2018). Previously it has been stated that skin metabolism, hair growth, sebum production, skin pH, and skin thickness differ between males and females. These differences may predispose male patients to PJI compared with female patients. (Badawy et al. 2017). Detailed preoperative patient counseling should take into account the increased PJI risk of male sex to manage modifiable surgery-related risks.

Younger age was not a PJI revision risk factor in our study, which gives support to some previous findings (Pedersen et al. 2010, Smith et al. 2018). Comorbidities are more common with older age and older age can thus affect the risk of developing PJI. However, Lenguerrand et al. (2018) stated recently based on the largest register study so far (623,253 THAs, 2,705 PJI revisions), that the PJI risk decreases with increasing age. The authors considered that their finding could be due to increased follow-up time compared with previous studies.

The correlation of obesity and risk of PJI is well documented in several prior studies and meta-analyses (Namba et al. 2012, Kunutsor et al. 2016, Kong et al. 2017, Kurtz et al. 2018, Lenguerrand et al. 2018, Smith et al. 2018, Triantafyllopoulos et al. 2018). Also, in our study BMI was associated with an increased risk of revision due to PJI. Patients with BMI of 30–35 and > 35 had a HR of 2.4 and 5.1, respectively. The PJI risk of those with BMI > 35 was even higher than that reported previously (OR 1.9 for BMI 35–40, OR 4 for BMI >40) (Smith et al. 2018). High BMI may be an even more prominent risk factor than assessed previously, special attention to which should be paid preoperatively. However, the effect of weight loss prior to THA on risk for PJI is not clear and more quality studies need to be done to clarify the subject (Lui et al. 2015, Li et al. 2019).

We found that long duration of operation was associated with an increased risk of revision for PJI for the first 3 postoperative weeks. This finding supports previous evidence (Engesaeter et al. 2006, Pedersen et al. 2010). Similar to our findings, Pedersen et al. (2010) have stated that duration of 2 hours or more increased PJI rate. On the other hand, Namba et al. (2012) found that duration of operation was not an independent risk factor for PJI. Specializing in THA increases the numbers performed, which probably decreases operation time. Unfortunately, our data did not include surgeon volume data. High hospital volume in our study was associated with increased PJI rate.

Bilateral operation was associated with increased risk of revision for PJI for the first 3 postoperative weeks; previous studies (Namba et al. 2012, Kong et al. 2017) have found the same association, but with no regard to time from operation. The risk of PJI should be considered in elective management of patients who require both hips to be operated on in the same operation.

We found that spinal anesthesia was associated with lower risk of PJI, whereas epidural anesthesia and general anesthesia were associated with increased risk of revision due to PJI in comparison with other anesthesia options. It has been stated earlier that neuraxial anesthesia is associated with decreased PJI rate compared with general anesthesia (Helwani et al. 2015, Johnson et al. 2016, Lenguerrand et al. 2018, Memtsoudis et al. 2019, Scholten et al. 2019). We are unaware of such data showing that epidural anesthesia would be associated with increased risk of revision due to PJI. Epidural anesthesia is often used in patients with anticipated longer operation time and hence might be associated with increased risk of complications.

Previous studies concerning bearing couples have found that ceramic-on-ceramic may be a protective factor for developing PJI (Lee et al. 2016, Pitto and Sedel 2016, Kurtz et al. 2017, Lenguerrand et al. 2018). Kurtz et al. (2017) stated that THA patients with ceramic-on-polyethylene and ceramic-on-ceramic bearings had reduced risk of infection relative to metal-on-polyethylene bearings (HR 0.9, HR 0.7 respectively). Lenguerrand et al. (2018) found that the risk of revision for PJI was influenced by the type of bearing couples and varied according to the time period. In the early postoperative period, no differences were observed. Ceramic-on-ceramic and ceramic-on-polyethylene surfaces were associated with a lower risk of long-term revision (from 12 months for ceramic-on-ceramic and 24 months for ceramic-on-polyethylene postoperation onwards) than metal-on-polyethylene bearings (Lenguerrand et al. 2018). Contrary to previous studies, we found that ceramic-on-ceramic was associated with a lower rate of revision for PJI in the early time period, as our study did not include long-term infections. Further, ceramic-on-UHXLPE did not protect against PJI in our study. It is likely that this finding is affected by residual confounding as ceramic-on-ceramic population differs from other surface groups regarding patient-related factors. A ceramic-on-ceramic bearing couple tends to be used in younger and healthier patients with less comorbidity. Also, the surgeons using ceramic-on-ceramic may be more experienced. This residual confounding likely affects the results even after adjusting.

Fixation method was not associated with PJI in our study. Previous reports have been contradictory. Lenguerrand et al. (2018) stated that in the early postoperative period patients who had undergone a cementless, hybrid, or reverse hybrid THA were at higher risk than those with cemented implants but from 3 to 24 months they were at lower or similar risk. Pedersen et al. (2010) found a tendency for increased risk of revision for patients who had received cemented THA without antibiotic and hybrid THA relative to patients with cementless implants. Kunutsor et al. (2019) stated in the meta-analysis that, in the first six months, cementless fixations were associated with increased PJI risk when compared with cemented fixation. Overall cemented fixation was associated with an increased PJI risk compared with uncemented THA. Most PJIs occur during the first postoperative year, and it seems that bone cement may protect fragile patients with cemented THAs from early infections. Antibiotics in the bone cement are not released later on, so the protective effect finishes. The fixation method is a variable that might be affected by both known and unknown confounders. For example, elderly patients are prone to have a cemented THA. On the other hand, antibiotics in the bone cement may protect from PJI. Surgical approach did not have an effect on PJI risk in the current study whereas previous studies have been inconclusive on the subject (Namba et al. 2012, Lenguerrand et al. 2018, Smith et al. 2018, Triantafyllopoulos et al. 2018).

High-volume hospital was associated with increased risk of revision, though preceding evidence has been contradictory. In study from the United States, no association between higher volume hospitals (> 200 THAs annually) and PJI revisions (Namba et al. 2012) was found. However, in a study from the UK risk of early infections was increased in THAs undertaken in high-volume hospitals (> 255 THAs in the previous 12 months) (Lenguerrand et al. 2018). In our study previous contributing operation was associated with increased risk of PJI in univariable analysis and a similar association has been presented before (Cordero-Ampuero and De Dios 2010).

Previous reports concerning preoperative diagnosis and PJI risk after primary THA have often found an association (Pedersen et al. 2010, Namba et al. 2012, Lenguerrand et al. 2018). In our study, “other” preoperative diagnosis vs. primary osteoarthritis was associated with higher risk of PJI. Conditions that cause, e.g., avascular necrosis, such as steroid use or irradiation, cause immunosuppression and also predispose towards PJI.

We acknowledge that our study has several limitations. Although prospectively collected, our data is observational. Further, FAR does not incorporate comprehensive data on possibly relevant patient-related factors such as socioeconomic status, smoking status, or comorbidities, although ASA class is a crude estimate of medical condition. Even though FAR has included new variables since 2014 there still might be some confounding factors not included in the FAR influencing our results, such as the lower risk of infection in ceramic-on-ceramic articulations. Furthermore, completeness of revision surgery of FAR is 81% compared with discharge register so we are missing some PJI revisions (FAR 2016). Those revision operations performed on call (PJI, fractures, dislocations) are probably slightly underreported compared with elective revisions (wear, metallosis). However, we do not think that this causes serious bias to our results. The mortality rate was low and thus we considered death not to be a significant competing event with PJI revision. The PJI diagnosis reflects a clinical judgment sufficient to lead the surgeon to conduct a revision operation. Our data is recorded in operating theatres based on clinical diagnosis, and is not complemented afterwards based on, e.g., microbiology data, which may lead to underestimation of the incidence of PJIs. The strength of this study is a large, unselected population-based register setting with prospectively collected data.

In summary we found that high BMI, advanced ASA class, bleeding over 500 mL and the use of epidural and general anesthesia increased the risk of revision for PJI, whereas ceramic-on-ceramic bearing couple and spinal anesthesia decreased revision risk. Simultaneous bilateral operation and duration of operation over 120 minutes increased the risk of revision for PJI during the first 3 postoperative weeks.

## Supplementary Material

Supplemental Material

## Supplementary data

Tables 1 and 3 are available as supplementary data in the online version of this article, http://dx.doi.org/10.1080/17453674.2021.1944529

All authors participated in designing the protocol. MV and LE performed statistical analyses. All the authors participated in interpreting the results, and writing and revision of the manuscript. All the authors read and approved the final manuscript.

*Acta* thanks Ove Furnes and Anna Stefánsdóttir for help with peer review of this study.
