# Supplementary material for: Risk factors for prosthetic joint infections following total hip arthroplasty based on 33,337 hips in the Finnish Arthroplasty Register from 2014 to 2018
Source: Acta Orthop. 2021 Jul 1;92(6):665–72. doi: 10.1080/17453674.2021.1944529 (PMC8635657; doi:10.1080/17453674.2021.1944529)
Supplement: Supplemental Material [file IORT_A_1944529_SM7359.pdf]

Supplementary data

Table 1. Demographic data

Characteristic	All n (%) n = 33,337	Infection n (%) n = 350	No infection n (%) n = 32,987
Age, n	33,330	350	32,980
≤ 55	4,507 (14)	47 (13)	4,460 (14)
56–65	8,333 (25)	92 (26)	8,241 (25)
66–75	12,399 (37)	122 (35)	12,277 (37)
≥ 76	8,091 (24)	89 (25)	8,002 (24)
Sex, n	33,319	350	32,969
Male	14,317 (43)	198 (57)	14,119 (43)
Female	19,002 (57)	152 (43)	18,850 (57)
Operated side	33,337	350	32,987
Right	18,500 (56)	181 (52)	18,319 (56)
Left	14,837 (45)	169 (48)	14,668 (45)
Simultaneous bilateral operation			
n	33,337	350	32,987
No	32,425 (97)	337 (96)	32,088 (97)
Yes	912 (2.7)	13 (3.7)	899 (2.7)
ASA physical status classification			
n	32,697	346	32,351
ASA I	4,013 (12)	22 (6.4)	3,991 (12)
ASA II	16,117 (49)	152 (44)	15,965 (49)
ASA III–IV	12,567 (38)	172 (50)	12,395 (38)
BMI, n	30,382	339	30,043
≤ 20	716 (2.3)	3 (0.9)	713 (2.4)
21–25	7,715 (25)	49 (14)	7,666 (26)
26–30	12,450 (41)	102 (30)	12,348 (41)
31–35	6,832 (23)	102 (30)	6,730 (22)
> 35	2,669 (8.8)	83 (25)	2,586 (8.6)
Preoperative diagnosis			
n	32,315	346	31,969
Primary osteoarthritis	27,965 (87)	285 (82)	27,680 (87)
Fracture	1,366 (4.2)	14 (4.0)	1,352 (4.2)
Inflammatory arthritis	591 (1.8)	8 (2.3)	583 (1.8)
Other	2,393 (7.4)	39 (11)	2,354 (7.4)
Hospital volume, n	33,333	350	32,983
Low (< 240)	13042 (39)	123 (35)	12,919 (39)
Medium (240–480)	10,279 (31)	106 (30)	10,173 (31)
High (> 480)	10,012 (30)	121 (35)	9,891 (30)
Level of education (surgeon)			
n	29,853	329	29,524
Orthopedic specialist	28,438 (95)	314 (95)	28,124 (95)
Resident	1,415 (4.7)	15 (4.6)	1,400 (4.7)
Level of education (assistant)			
n	29,003	316	28,687
Orthopedic specialist	2,877 (9.9)	35 (11)	2,842 (9.9)
Resident	8,162 (28)	102 (32)	8,060 (28)
No	1,189 (4.1)	13 (4.1)	1,176 (4.1)
Other	16,775 (58)	166 (53)	16,609 (58)
Surgical approach	32,652	349	32,303
Anterolateral (modified Hardinge)	6,151 (19)	64 (18)	6,087 (19)
Posterior	26,203 (80)	281 (81)	25,922 (80)
Anterior (Smith-Peterson)	298 (0.9)	4 (1.2)	294 (0.9)
Intraoperative bleeding	31,381	339	31,042
< 500 mL	21,839 (70)	204 (60)	21,635 (70)
> 500 mL	9,542 (30)	135 (40)	9,407 (30)
Duration (minutes)	27,645	312	27,333
< 45	1,987 (7.2)	17 (5.4)	1,970 (7.2)
45–59	5,045 (18)	39 (13)	5,006 (18)
60–89	13,498 (49)	151 (48)	13,347 (49)
90–120	5,404 (20)	71 (23)	5,333 (20)
> 120	1,711 (6.2)	34 (11)	1,677 (6.2)
Anesthesia (spinal)	32,604	347	32,257
No	2,485 (7.6)	43 (12)	2,442 (7.6)
Yes	30,119 (92)	304 (88)	29,815 (92)

Characteristic	All n (%) n = 33,337	Infection n (%) n = 350	No infection n (%) n = 32,987
Anesthesia (epidural), n	32,604	347	32,257
No	31,813 (98)	329 (95)	31,484 (98)
Yes	791 (2.4)	18 (5.2)	773 (2.4)
Anesthesia (general), n	32,604	347	32,257
No	30,072 (92)	305 (88)	29,767 (92)
Yes	2,532 (7.8)	42 (12)	2,490 (7.7)
Anesthesia (nerve block)	32,604	347	32,257
No	32,598 (100)	347 (100)	32,251 (100)
Yes	6 (0.0)	0 (0.0)	6 (0.0)
Anesthesia (LIA), n	32,604	347	32,257
No	26,367 (81)	286 (82)	26,081 (81)
Yes	6,237 (19)	61 (18)	6,176 (19)
Complications during surgery (fracture)			
n	31,395	336	31,059
No	30,993 (99)	332 (99)	30,661 (99)
Yes	402 (1.3)	4 (1.2)	398 (1.3)
Previous contributing operations			
n	28,071	315	27,756
No	27,466 (98)	303 (96)	27,163 (98)
Yes	605 (2.2)	12 (3.8)	593 (2.1)
Antibiotic prophylaxis, n	32,898	349	32,549
Cefuroxime	31,115 (95)	337 (97)	30,778 (95)
Clindamycin	1,043 (3.2)	6 (1.7)	1,037 (3.2)
Vancomycin	81 (0.2)	0 (0.0)	81 (0.2)
Other ab prophylaxis	585 (1.8)	6 (1.7)	579 (1.8)
Not used	74 (0.2)	0 (0.0)	74 (0.2)
Antithrombotic prophylaxis			
n	32,633	347	32,286
Enoxaparin	23,874 (73)	266 (77)	23,608 (73)
Rivaroxaban	5,808 (18)	50 (14)	5,758 (18)
Tinzaparin	1,308 (4.0)	9 (2.6)	1,299 (4.0)
Warfarin	103 (0.3)	3 (0.9)	100 (0.3)
Other	1,230 (3.8)	9 (2.6)	1,221 (3.8)
Not used	310 (0.9)	10 (2.9)	300 (0.9)
Anticoagulants, n	31,873	346	31,527
Tranexamic acid	28,703 (90)	314 (91)	28,389 (90)
No	2,856 (9.0)	25 (7.2)	2,831 (9.0)
Other	314 (1.0)	7 (2.0)	307 (1.0)
Mechanical antithrombotic prophylaxis			
n	27,137	294	26,843
Not used	15,873 (59)	164 (56)	15,709 (59)
Calf muscle pump	61 (0.2)	2 (0.7)	59 (0.2)
Surgical stocking	11,203 (41)	128 (44)	11,075 (41)
Antimicrobial incise drape			
n	8,818	84	8,734
No	426 (4.8)	4 (4.8)	422 (4.8)
Yes	8,392 (95)	80 (95)	8,312 (95)
Fixation, n	30,150	312	29,838
Cementless	18,655 (62)	190 (61)	18,465 (62)
Cemented	3,008 (10)	29 (9.3)	2,979 (10)
Hybrid	6,837 (23)	79 (25)	6,758 (23)
Reverse hybrid	1,650 (5.4)	14 (4.5)	1,636 (5.5)
Bearing couple, n	25,107	255	24,852
Metal-on-UHXLPE	12,652 (50)	152 (60)	12,500 (50)
Ceramic-on-ceramic	2,786 (11)	13 (5.1)	2,773 (11)
Ceramic-on-UHXLPE	7,063 (28)	72 (28)	6,991 (28)
Ceramized metal-on-UHXLPE	1,445 (5.8)	16 (6.3)	1,429 (5.7)
Other	1,161 (4.6)	2 (0.8)	1,159 (4.7)
Femoral head size (mm)	32,452	342	32,110
28	347 (1.1)	6 (1.8)	341 (1.1)
32	7,836 (24)	49 (14)	7,787 (24)
36	23,958 (74)	283 (83)	23,675 (74)
>36	311 (1.0)	4 (1.2)	307 (1.0)

Acta Orthopaedica 2021; 92 (DOI: 10.1080/17453674.2021.1944529)

Table 3. Univariable analysis of possible risk factors for revision for PJI

Variable	Hazard ratio (95% CI)
Age (reference ≤ 55)	
56–65	1.1 (0.7–1.5)
66–75	0.9 (0.7–1.3)
> 76	1.1 (0.7–1.5)
Sex (reference male)	
Female	0.6 (0.5–0.7)
Operated side (reference right)	
Left	1.2 (0.9–1.4)
ASA physical status classification (reference ASA I)	
ASA II	1.7 (1.1–2.7)
ASA III–IV	2.5 (1.6–3.9)
BMI (reference BMI 21–25)	
≤ 20	0.7 (0.2–2.1)
26–30	1.3 (0.9–1.8)
31–35	2.3 (1.7–3.3)
> 35	5.0 (3.5–7.1)
Preoperative diagnosis (reference primary osteoarthritis)	
Fracture	1.0 (0.6–1.7)
Inflammatory arthritis	1.3 (0.7–2.7)
Other	1.6 (1.1–2.2)
Hospital volume (reference low (< 240))	
Medium (240–480)	1.1 (0.9–1.4)
High (> 480)	1.3 (1.0–1.7)
Level of education (surgeon) (reference orthop. specialist)	
Resident	1.0 (0.6–1.6)
Level of education (assistant) (reference orthop. specialist)	
Resident	1.0 (0.7–1.5)
No	0.9 (0.5–1.7)
Other	0.8 (0.6–1.2)
Surgical approach (reference anterolateral (modified Hardinge))	
Posterior	1.1 (0.8–1.4)
Anterior (Smith-Peterson)	1.3 (0.5–3.6)
Intraoperative bleeding (reference < 500 mL)	
> 500 ml	1.5 (1.2–1.9)
Anesthesia (spinal) (reference no)	
Yes	0.6 (0.4–0.8)
Anesthesia (epidural) (reference no)	
Yes	2.2 (1.4–3.5)
Anesthesia (general) (reference no)	
Yes	1.7 (1.2–2.3)
Previous contributing operations (reference no)	
Yes	1.8 (1.0–3.2)
Antibiotic prophylaxis (reference cefuroxime)	
Clindamycin	0.5 (0.2–1.2)
Other antibiotic prophylaxis	0.9 (0.4–2.0)
Vancomycin	0.0 (0.0–Inf)
Not used	0.0 (0.0–Inf)
Antithrombotic prophylaxis (reference enoxaparin)	
Warfarin	2.7 (0.9–8.4)
Rivaroxaban	0.8 (0.6–1.0)
Tinzaparin	0.6 (0.3–1.2)
Not used	2.8 (1.5–5.3)
Other	0.6 (0.3–1.2)
Anticoagulant medications (reference tranexamic acid)	
No	0.8 (0.5–1.2)
Other	2.0 (0.9–4.2)
Mechanical antithrombotic prophylaxis (reference not used)	
Calf muscle pump	3.0 (0.8–12.3)
Surgical stocking	1.1 (0.9–1.4)
Antimicrobial incise drape (reference not used)	
Yes	1.0 (0.4–2.8)
Fixation (reference cementless)	
Cemented	1.0 (0.6–1.4)
Hybrid	1.1 (0.9–1.5)
Reverse hybrid	0.9 (0.5–1.5)
Bearing couple (reference metal-on-UHXLPE)	
Ceramic-on-ceramic	0.4 (0.2–0.7)
Ceramic-on-UHXLPE	0.9 (0.6–1.1)
Ceramized metal-on-UHXLPE	0.9 (0.5–1.5)
Other	0.1 (0.0–0.6)
Femoral head size (reference 32 mm)	
28 mm	2.8 (1.2–6.5)
36 mm	1.9 (1.4–2.6)
>36	2.1 (0.7–5.7)

LIA = local infiltrative anesthesia,
UHXLPE = ultra-highly crosslinked polyethylene.
